# Brain network of athletes in motor imagery and action anticipation: an ALE meta-analysis and MACM analysis

**DOI:** 10.3389/fspor.2025.1652165

**Published:** 2025-08-25

**Authors:** Yanqiu Wang, Yang Sun, Jiangsheng Yu, Xiaorong Cheng, Zhebin Niu

**Affiliations:** ^1^ School of Physical Education and Sports, Central China Normal University, Wuhan, China; ^2^ School of Psychology, Central China Normal University, Wuhan, China; ^3^ Faculty of Physical Education, China West Normal University, Nanchong, China

**Keywords:** action anticipation, motor imagery, athletes, neuroimaging, activation likelihood estimation, meta-analytic connectivity modeling

## Abstract

Understanding how athletes mentally simulate and anticipate actions provides key insights into experience-driven brain plasticity. While previous studies have investigated motor imagery and action anticipation separately, little is known about how their underlying neural mechanisms converge or diverge in expert performers. This study conducted a meta-analysis using activation likelihood estimation (ALE) and meta-analytic connectivity modeling (MACM) to compare brain activation patterns between athletes and non-athletes across both tasks. We systematically reviewed functional magnetic resonance imaging studies and included 20 eligible studies. ALE was used to identify consistent activation patterns, followed by contrast and conjunction analyses. MACM was used to further explored connectivity among key brain regions. Results showed that athletes exhibited stronger activation in the left middle and precentral gyrus during motor imagery, and in the superior frontal gyrus, bilateral precentral gyrus, and right middle frontal gyrus during action anticipation. Non-athletes showed greater activation in visual regions such as the middle occipital gyrus. Connectivity analyses revealed that athletes formed a cohesive fronto-parietal-temporal network integrating motor imagery and action prediction, which was not observed in non-athletes. These findings highlight a nested and efficient action observation network in athletes, supporting the integration of internally generated and externally guided action processes. This work advances models of perceptual-motor expertise and provides insight into how long-term sports training modulates brain plasticity.

## 1 Introduction

In recent years, the integration of neuroscience and performance science has opened new avenues for understanding expert behavior in domains such as sport and dance. A growing body of research has demonstrated that long-term training induces both functional and structural brain changes, particularly in tasks that require precise coordination between perception and action (1–3). Within this framework, athletes represent a unique population for studying experience-dependent neural plasticity (4). Two perceptual-motor processes—motor imagery and action anticipation—have gained particular attention, as they reflect distinct yet complementary cognitive operations underlying expert performance (5–7).

Motor imagery refers to the internal simulation of movements without overt physical execution, engaging internal models based on previously encoded sensorimotor experiences (8, 9). In contrast, action anticipation involves externally driven processes that rely on perceiving environmental cues and predicting others’ behaviors in real time (10, 11). Both tasks are essential for athletes, who often mentally rehearse complex sequences (e.g., gymnastics routines) and anticipate an opponent's next move (e.g., in tennis or football). Behavioral studies consistently show that experts outperform novices in these tasks, due to their enhanced ability to extract kinematic information and generate predictive models (12, 13).

Neuroimaging research reveals that athletes engage specialized brain networks during motor imagery and action anticipation, particularly regions within the action observation network (AON) and mirror neuron system (MNS) (14, 15). The AON broadly encompasses the MNS, with both systems sharing key brain regions such as inferior frontal gyrus (IFG), premotor cortex, and inferior parietal lobule, but the AON includes additional areas and processes beyond the classic mirror neuron framework (16). The neural efficiency hypothesis suggests that experts utilize these networks more effectively, exhibiting reduced and more focused cortical activation while maintaining high performance (17–19). While these findings underscore the role of AON/MNS in expert behavior, prior studies have largely examined motor imagery or action anticipation in isolation, limiting our understanding of how these processes differ or converge in the expert brain.

This separation in the literature has led to a fragmented understanding of the shared and distinct neural mechanisms underlying motor imagery and action anticipation. Many studies rely on passive observation or variable task designs and sample characteristics, which complicates direct comparisons (20–22). Moreover, while meta-analyses have identified activation patterns in action observation (23) and action anticipation (24, 25), they rarely contrast internally imaginary vs. external motor response processes within a unified framework (26). As a result, the specific neurocognitive architecture that differentiates motor imagery and action anticipation in expert athletes remains poorly defined.

The present study aims to fill this gap by systematically comparing brain activation patterns in athletes during motor imagery and action anticipation tasks using meta-analytic techniques. Drawing on methods such as activation likelihood estimation (ALE) and meta-analytic connectivity modeling (MACM), this study seeks to identify not only shared neural substrates but also task-specific spatially distinct or nested activation patterns across these two expert-related domains. Conducting these analyses, we first assumed that athletes, compared to novices, would exhibit greater activation in regions such as the AON/MNS. We second hypothesized that athletes would exhibit a more efficient brain activation network between motor imagery and action anticipation. These findings are expected to refine theoretical models of perceptual-motor expertise and support the development of precision neurocognitive interventions for optimizing motor training, enhancing skill acquisition, and informing rehabilitation strategies in both athletic and clinical contexts.

## 2 Methods

### 2.1 Literature search

A systematic review was conducted of relevant articles published in the Web of Science, EBSCO, and PubMed databases before July 22, 2025. The keywords were set as (“sport expertise” OR “motor expertise” OR “skill expertise” OR “expert” OR “player” OR “athlete”) and (“fMRI” OR “functional magnetic resonance imaging” OR “neuroimaging” OR “brain” OR “cortical” OR “neural”). A total of 14,604 articles were retrieved. The data extracted included study characteristics, participant information, task types, and imaging outcomes. This process followed the literature selection methods recommended by the Preferred Reporting Items for Systematic Reviews and Meta-Analyses (PRISMA) guidelines.

### 2.2 Study selection

The inclusion criteria for the study were as follows:

(1) The research subjects were athletes (professional athletes/professional college athletes or the average training years exceeds 10 years), with no restrictions on sport type, gender;
(2) Only studies that included both athletes and control groups, or studies that compared brain activation regions between athletes and control groups, were included;
(3) To analyze brain activation, studies using imaging techniques such as functional magnetic resonance imaging (fMRI), positron emission tomography (PET), and single-photon emission computed tomography (SPECT) were initially selected. To ensure that all original data in the calculations had approximate spatial resolution, only fMRI data were ultimately included;
(4) Studies with clear motor tasks were selected, including action anticipation (e.g., predicting tennis ball landing spots) and motor imagery (e.g., imagining diving);
(5) Study results: Studies that used 3D standard coordinates in Talairach space and MNI (Montreal Neurological Institute) space for whole-brain data analysis of activation points were included.

Studies were excluded if they met any of the following criteria:

(1) Protocols, abstracts, review articles, or case reports;
(2) Duplicate articles or overlapping themes;
(3) Studies unrelated to athletes;
(4) Studies focusing on structural imaging, resting-state, or functional brain connectivity;
(5) Studies focusing on region-of-interest (ROI) analysis;
(6) Studies including data from only athletes or only control groups;
(7) Studies using TMS (Transcranial Magnetic Stimulation), MEG (Magnetoencephalograp), or EEG (Electroencephalography) data.

### 2.3 Activation likelihood estimation

In this study, GingerALE 3.0.2 (http://brainmap.org/ale) was used for meta-analysis (27, 28). As a coordinate-based meta-analysis tool, it was necessary to extract reported activation coordinates from the literature included in the meta-analysis before data analysis. The coordinates include brain regions activated by athletes and non-athletes during tasks, as well as brain regions showing activated contrasts between the two groups during tasks. Each task involved three databases, which were analyzed separately. Since the meta-analysis was conducted in the MNI standard space, Talairach space were transformed into MNI standard space using the conversion tool provided in the software. According to a previous work (29), an uncorrected significance threshold of *p* < 0.001 was adopted to control Type I errors, with a minimum volume set to 250 mm^3^. Results were reported using Mango (http://ric.Uthscsa.edu/mango/) and Brainnet Viewer (30).

### 2.4 Contrast and conjunction analyses

Statistical comparisons of ALE maps obtained from single meta-analyses were performed using the ALE method. Specifically, the analyses included: (1) motor imagery: brain regions commonly activated in athletes and non-athletes; (2) action anticipation: brain regions commonly activated in athletes and non-athletes; (3) differences and commonalities in brain activation mechanisms between non-athletes and athletes during action anticipation and motor imagery. To identify stage-specific patterns, permutation tests will be performed and parameters adjusted to enhance sensitivity to differences while maintaining a certain level of rigor. Specifically, contrast and conjunction analyses were conducted using the following parameters: uncorrected *p* < 0.05, a minimum cluster size exceeding 150 mm^3^, and 10,000 permutations (31).

### 2.5 MACM analyses

ROIs with a radius of 10 mm were created based on the results of meta-analysis. Two ROIs were defined for brain regions significantly activated in athletes compared to non-athletes during the motor imagery task. Four ROIs were defined for brain regions significantly activated in athletes compared to non-athletes during the action anticipation task. Two ROIs were defined for brain regions commonly activated in both athletes and non-athletes during the action anticipation task. Five ROIs were defined for brain regions significantly more activated in athletes during the action anticipation task than during the motor imagery task. Two ROIs were defined for brain regions significantly more activated in non-athletes during the action anticipation task than during the motor imagery task. Two ROIs were defined for brain regions significantly more activated in non-athletes during the motor imagery task than during the action anticipation task.

Network modeling for MACM analysis was conducted using methods consistent with prior research (32–35). To summarize this procedure, Mango was used to visualize the uncorrected MACM overlay for each seed coordinate on an MNI template (Colin27_T1_seg_MNI.nii). GingerALE was employed for meta-analysis of the activated coordinates, with parameters set at uncorrected *p* < 0.001 and minimum volume set to 250 mm^3^ (31). The uncorrected *p*-values for meta-analytic connectivity were extracted and recorded for each seed region and all other specified nodes.

The *p*-values for multiple comparisons between nodes were corrected using a Bonferroni correction (*p* = 0.05/number of nodes). The corrected *p*-values represent the covariance statistics between nodes (i.e., each seed point used in MACM) and projections (i.e., connectivity between MACM seed points and other ROIs), which are used to generate edges in meta-analytic connectivity modeling. Connections between identified peak regions are mapped to display unidirectionality (arrows indicating unidirectional covariance), bidirectionality (bidirectional arrow indicating bidirectional covariance), or nodes with no significant connections to each other.

## 3 Results

### 3.1 Study selection and characteristics

**Figure 1:**
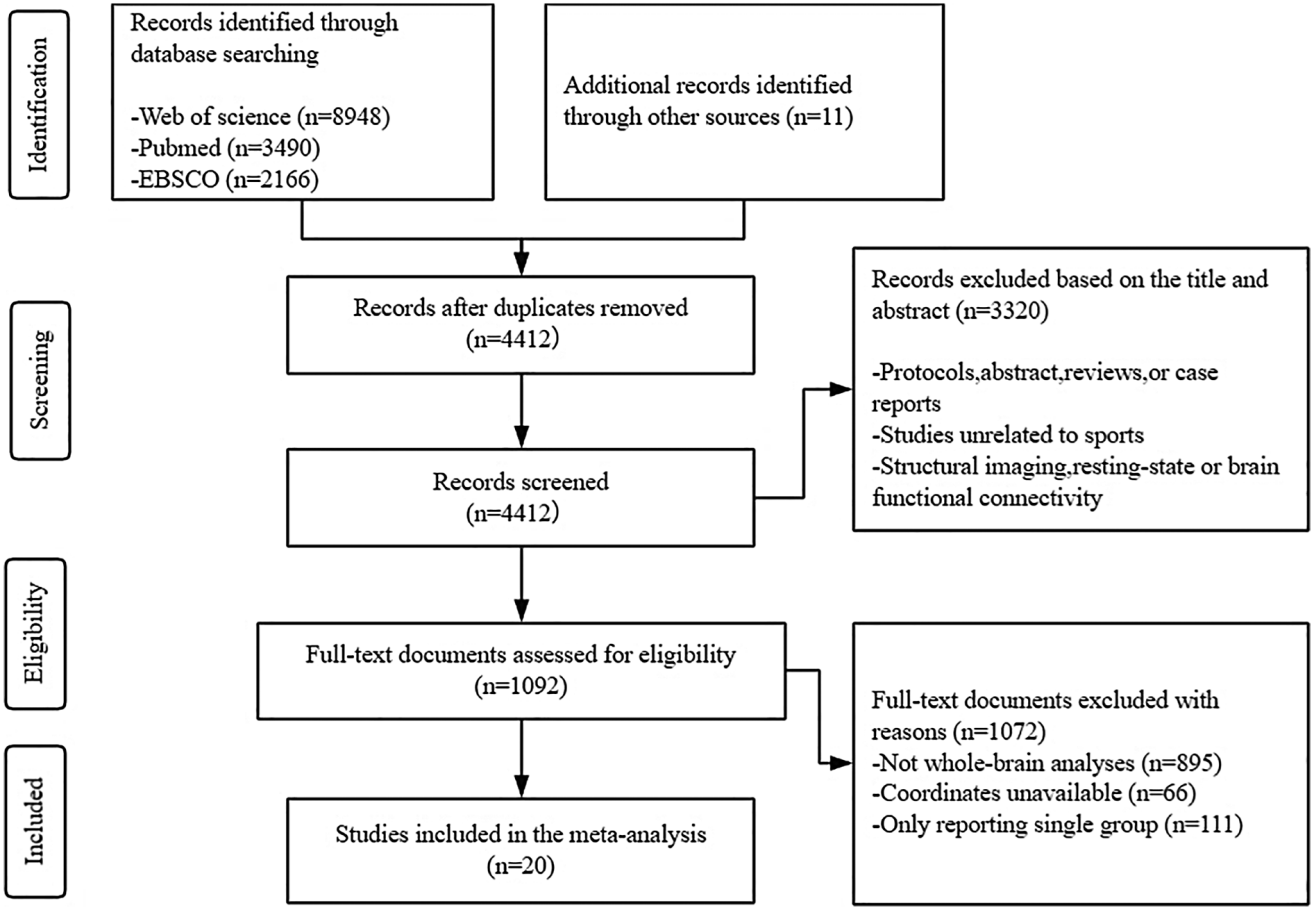
Flow diagram of the literature search used in this meta-analysis.

The literature database was queried, yielding 14,604 pertinent articles. After removing duplicates, 4,412 studies remained for screening. Of these, 3,320 were deemed irrelevant, resulting in a total of 1,093 eligible research articles. Subsequently, 1,072 studies were excluded for reasons including the absence of athlete or novice groups (*n* = 11), incomplete coordinate reporting (*n* = 66), and not whole-brain analyses (*n* = 895). Efforts to contact the corresponding authors of publications that lacked complete activation coordinates but met all other inclusion criteria received no responses. Consequently, 20 studies met the inclusion criteria and were included in the final meta-analysis, as depicted in the PRISMA flowchart (see Figure 1). Table 1 provides a comprehensive overview of all the included studies.

**Table 1:** Overview of the studies included in the meta-analysis.

Number	Study	Project	Athletes	Non-athletes	Athletes skill level	Space	Scanners	Voxel size (mm^3^)	Task type	Activation data
1	Wu et al. (36)	Basketball	15	15	PCA	MNI	3 T	3 mm slice thickness	Action Anticipation	Athletes/Non-athletes/Contrast
2	Xu et al. 2 (37)	Badminton	16	18	PA/PCA	MNI	3 T	/	Action Anticipation	Contrast
3	Wright et al. (38)	Football	17	17	PCA	MNI	3 T	3 mm slice thickness	Action Anticipation	Athletes/Non-athletes/Contrast
4	Wright et al. (13)	Badminton	8	8	PA/PCA	MNI	3 T	3 mm slice thickness	Action Anticipation	Contrast
5	Wimshurst et al. (39)	Field Hockey	15	15	PA	MNI	3 T	3 × 3 × 3	Action Anticipation	Athletes/Non-athletes/Contrast
6	Ji et al. (40)	Table Tennis	29	34	PCA	MNI	3 T	3.44 × 3.44 × 3.2	Action Anticipation	Contrast
7	Olsson and Lundström (41)	Ice Hockey	3	3	PA/AA	MNI	3 T	3.4 mm Slice thickness	Action Anticipation	Athletes/Non-athletes
8	Huang et al. (19)	Football	20	20	PCA	MNI	3 T	4.32 mm Slice thickness	Action Anticipation	Athletes/Non-athletes/Contrast
9	Meng et al. (42)	Volleyball	20	20	PCA	MNI	3 T	3.125 × 3.125 × 4	Action Anticipation	Athletes/Non-athletes/Contrast
10	Balser et al. (43)	Tennis	16	16	PA	MNI	1.5 T	5 mm slice thickness	Action Anticipation	Athletes/Non-athletes/Contrast
11	Abreu et al. (44)	Basketball	16	16	PA	MNI	3 T	3 × 3 × 3.8	Action Anticipation	Athletes/Non-athletes
12	Kim et al. (45)	Archery	8	8	ATY = 11.5	Talairach	3 T	/	Motor Imagery	Athletes/Non-athletes
13	Kim et al. (46)	Archery	12	14	ATY = 15.1	MNI	3 T	4 mm slice thickness	Motor Imagery	Athletes/Non-athletes/Contrast
14	Zhang et al. (47)	Basketball	24	25	PA/PCA	MNI	3 T	3.3 × 3.3 × 4	Motor Imagery	Athletes/Non-athletes/Contrast
15	Olsson et al. (48)	High Jump	12	12	PCA	MNI	1.5 T	4.4 mm slice thickness	Motor Imagery	Athletes/Non-athletes
16	Wei and Luo (49)	Diving	12	12	PA	Talairach	3 T	3.4 × 3.4 × 4.0	Motor Imagery	Athletes/Non-athletes
17	Chang et al. (50)	Archery	20	18	PA/PCA	MNI	3 T	4 mm slice thickness	Motor Imagery	Athletes/Non-athletes/Contrast
18	Zhang et al. (51)	Basketball	12	12	PCA	MNI	3 T	3.3 × 3.3 × 4	Motor Imagery	Athletes/Non-athletes/Contrast
19	Wang et al. (52)	Dance	24	24	PCA	MNI	3 T	3.44 × 3.44 × 3.2	Motor Imagery	Athletes/Non-athletes/Contrast
20	Kim et al. (53)	Archery	20	20	PA/PCA	MNI	3 T	4 mm slice thickness	Motor Imagery	Athletes/Non-athletes/Contrast

PA, professional athletes; PCA, professional college athletes; ATY, average training years.

### 3.2 Single activation analysis of athletes and non-athletes in different tasks

We analyzed the brain activation patterns of athletes and non-athletes during motor imagery and action anticipation separately.

#### 3.2.1 Neural activation during motor imagery tasks in athletes and non-athletes

**Figure 2:**
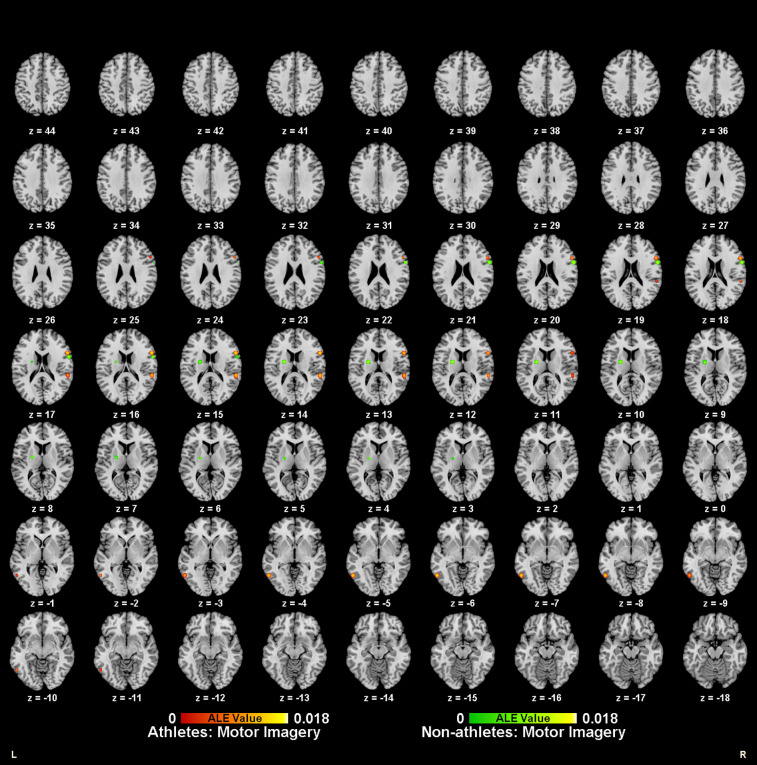
Neural activation during motor imagery tasks in athletes (red-yellow cluster) and non-athletes (green-yellow cluster).

Meta-analysis of brain activity in athletes during motor imagery tasks compared to baseline included nine studies, in which 144 athletes produced 95 activity increase points during task performance. Results showed a total of six activation clusters, concentrated in the left medial frontal gyrus (MedFG BA6), bilateral precentral gyrus (PreCG BA6/4), right inferior frontal gyrus (IFG BA44), left inferior temporal gyrus (ITG BA37), and right superior temporal gyrus (STG BA42) (Table 2, Figure 2).

**Table 2:** Neural activation during motor imagery tasks in athletes and non-athletes.

Cluster	Volume	Brain regions	Hemisphere	Brodmann area	MNI coordinates			ALE (×10^−2^)
					X	Y	Z	
Athletes: motor imagery								
1	1,512	Medial Frontal Gyrus	L	6	−6	−6	68	1.29
		Medial Frontal Gyrus	L	6	−4	−2	58	1.23
		Medial Frontal Gyrus	L	6	2	−4	68	1.16
		Superior Frontal Gyrus	R	6	12	0	68	0.90
2	864	Precentral Gyrus	L	6	−24	−12	54	1.78
3	800	Precentral Gyrus	R	4	20	−18	60	1.67
4	600	Inferior Frontal Gyrus	R	44	56	16	16	1.52
5	480	Inferior Temporal Gyrus	L	37	−56	−60	−6	1.53
6	400	Superior Temporal Gyrus	R	42	58	−36	14	1.34
Non-athletes: motor imagery								
1	1,648	Medial Frontal Gyrus	L	6	−8	2	62	1.82
2	536	Lentiform Nucleus	L	/	−22	−4	12	1.16
		Lentiform Nucleus	L	/	−22	−10	4	0.88
3	424	Medial Frontal Gyrus	R	6	4	2	68	1.22
4	384	Inferior Frontal Gyrus	R	9	60	6	20	1.33
5	312	Inferior Parietal Lobule	L	40	−36	−48	50	1.03
6	264	Precuneus	R	7	26	−66	58	0.99

Meta-analysis of brain activity in non-athletes during motor imagery compared to baseline tasks included nine studies, in which 145 participants produced 91 activity increase points during task performance. Results revealed six activation clusters, primarily in the bilateral medial frontal gyrus (MedFG BA6), left lentiform nucleus, right inferior frontal gyrus (IFG BA9), left inferior parietal lobule (IPL BA40), and right precuneus (Precuneus BA7) (Table 2, Figure 2).

#### 3.2.2 Neural activation during action anticipation tasks in athletes and non-athletes

**Figure 3:**
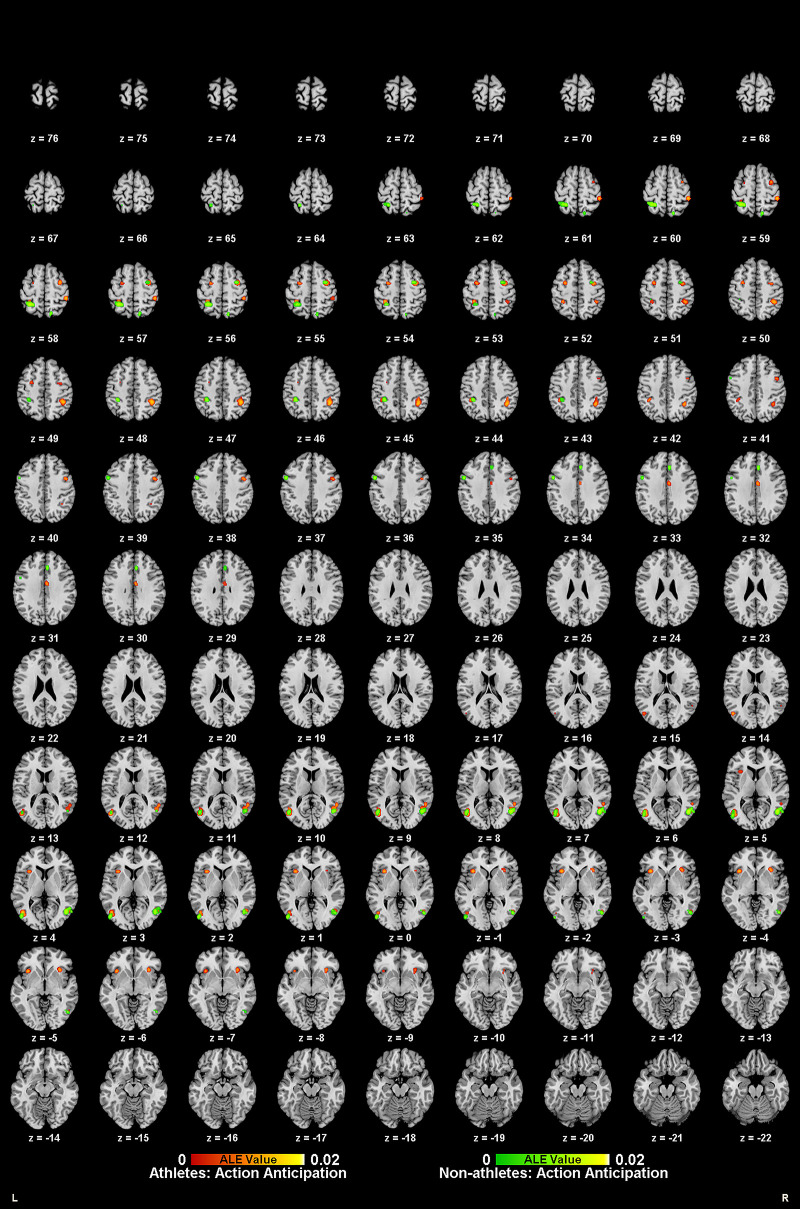
Neural activation during action anticipation tasks in athletes (red-yellow cluster) and non-athletes (green-yellow cluster).

Meta-analysis of brain activity in athletes during action anticipation tasks included eight studies, in which 122 athletes produced 124 activity increase points during task performance. Results showed a total of eleven activation clusters, concentrated in the left middle occipital gyrus (MOG BA37), right middle temporal gyrus (MTG BA37), right inferior parietal lobule (IPL BA40), bilateral precentral gyrus (PreCG BA6), bilateral claustrum, left precuneus (Precuneus BA7), and right cingulate gyrus (Cingulate Gyrus BA24) (Table 3, Figure 3).

**Table 3:** Neural activation during action anticipation tasks in athletes and non-athletes.

Cluster	Volume	Brain regions	Hemisphere	Brodmann area	MNI coordinates			ALE (×10^−2^)
					X	Y	Z	
Athletes: action anticipation								
1	1,656	Middle Occipital Gyrus	L	37	−46	−70	8	1.99
2	1,624	Middle Temporal Gyrus	R	37	48	−64	8	1.59
		Superior Temporal Gyrus	R	39	54	−52	10	1.11
3	1,224	Inferior Parietal Lobule	R	40	40	−46	48	1.56
4	712	Precentral Gyrus	R	6	34	−6	54	1.33
5	704	Claustrum	L	/	−34	18	−2	1.34
6	496	Claustrum	R	/	32	22	−6	1.10
		Lentiform Nucleus	R	/	30	14	−10	0.84
7	464	Precuneus	L	7	−32	−44	54	1.31
8	416	Inferior Parietal Lobule	R	40	46	−38	58	1.45
9	368	Precentral Gyrus	L	6	−26	−6	52	1.10
		Middle Frontal Gyrus	L	6	−28	−4	46	0.87
10	272	Precentral Gyrus	R	6	44	4	40	1.07
11	256	Cingulate Gyrus	R	24	4	−6	32	1.10
Non-athletes: action anticipation								
1	1,608	Middle Occipital Gyrus	R	37	46	−66	6	1.57
		Inferior Temporal Gyrus	R	37	50	−68	−2	1.09
		Inferior Temporal Gyrus	R	37	58	−68	4	0.90
2	1,208	Precuneus	L	7	−30	−50	58	1.54
3	856	Inferior Temporal Gyrus	L	19	−48	−76	2	1.21
		Middle Temporal Gyrus	L	37	−46	−66	8	1.13
4	384	Inferior Parietal Lobule	L	40	−34	−40	46	1.28
5	360	Precentral Gyrus	L	6	−52	6	36	1.26
6	272	Cingulate Gyrus	R	32	6	26	32	1.07
7	256	Precuneus	R	7	14	−70	58	1.02

Meta-analysis of brain activity in non-athletes during action anticipation tasks included eight studies, in which 122 non-athletes produced 119 activity increase points during task performance. Results revealed seven activation clusters, primarily in the right middle occipital gyrus (MOG BA37), bilateral precuneus (Precuneus BA7), left inferior temporal gyrus (ITG BA19), left inferior parietal lobule (IPL BA40), left precentral gyrus (PreCG BA6), and right cingulate gyrus (Cingulate Gyrus BA32) (Table 3, Figure 3).

### 3.3 Conjunction and contrast analyses between athletes and non-athletes

Pairwise conjunction and contrast analyses of brain activity were performed between athletes and non-athletes.

#### 3.3.1 Comparison between athletes and non-athletes during motor imagery

**Figure 4:**
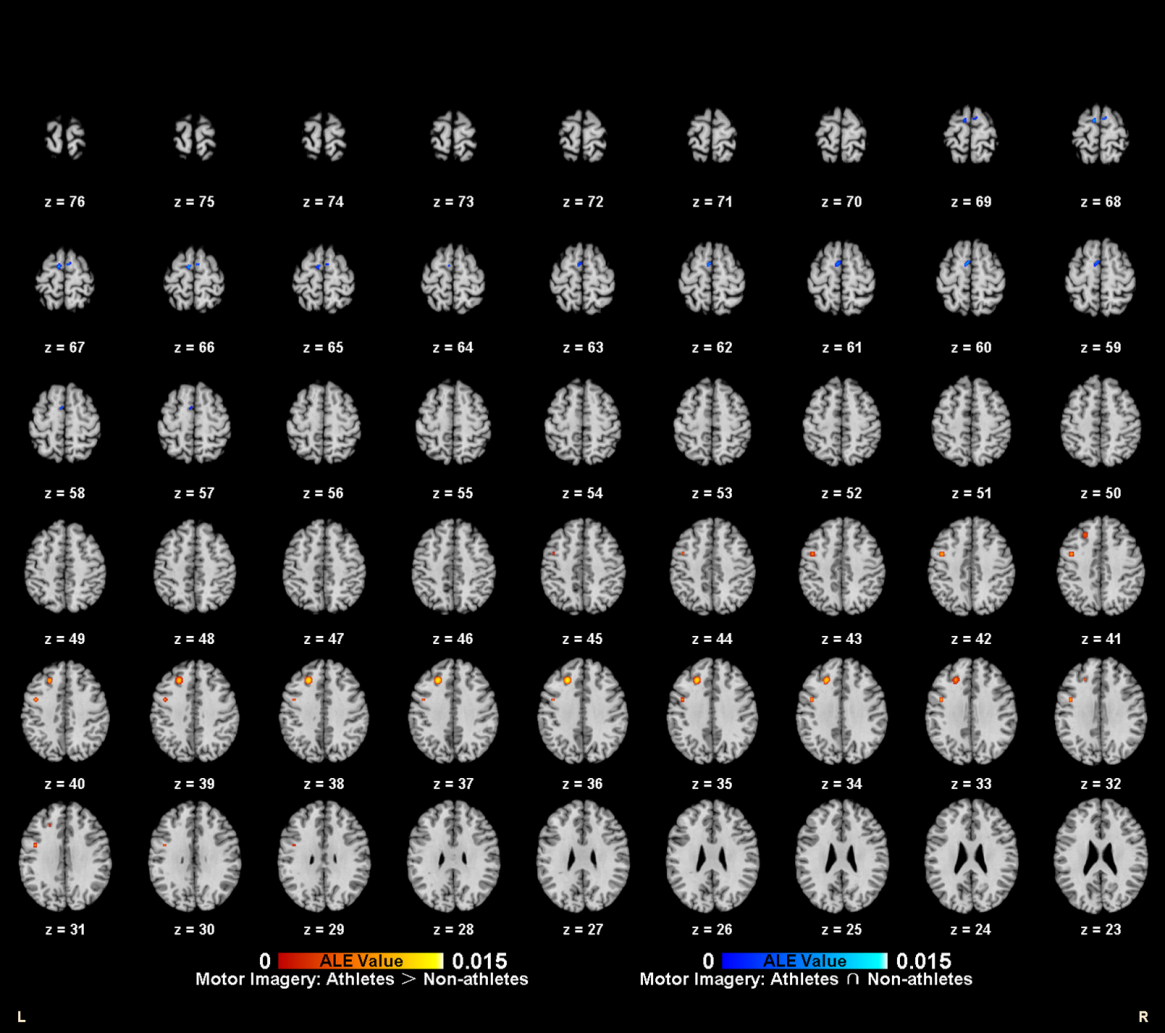
Common and specific brain activation in athletes and non-athletes during motor imagery. Red-yellow marks indicated clusters where athletes exhibited greater activation than non-athletes. Blue-cyan marks indicated clusters where athletes and non-athletes had overlapped activation.

Meta-analytic calculations of 95 reported brain region coordinates with significantly stronger activation in athlete groups across five studies identified two significant activation clusters, concentrated in the left middle frontal gyrus (MFG BA8) and left precentral gyrus (PreCG BA6) (Table 4, Figure 4).

**Table 4:** Conjunction and contrast analyses between athletes and non-athletes during motor imagery.

Cluster	Volume	Brain regions	Hemisphere	Brodmann area	MNI coordinates			ALE (×10^−2^)
					X	Y	Z	
Motor imagery: athletes > non-athletes								
1	560	Middle Frontal Gyrus	L	8	−22	28	36	1.47
2	272	Precentral Gyrus	L	6	−42	0	42	0.93
Motor imagery: non-athletes > athletes								
-	-	-	-	-	-	-	-	-
Motor imagery: athletes ∩ non-athletes								
1	232	Medial Frontal Gyrus	L	6	−8	−6	66	1.02
		Medial Frontal Gyrus	L	6	−6	0	60	0.96

Meta-analytic calculations of 69 reported brain region coordinates, with significantly stronger activation in control groups across four studies, found no activation clusters in the control group brain activity (Table 4).

To determine common activation regions between athletes and non-athletes during motor imagery tasks, a conjunction analysis was performed on the individual ALE results of the two groups. Findings showed common activation of the left medial frontal gyrus (MedFG BA6) in both groups (Table 4, Figure 4).

#### 3.3.2 Comparison between athletes and non-athletes during action anticipation

**Figure 5:**
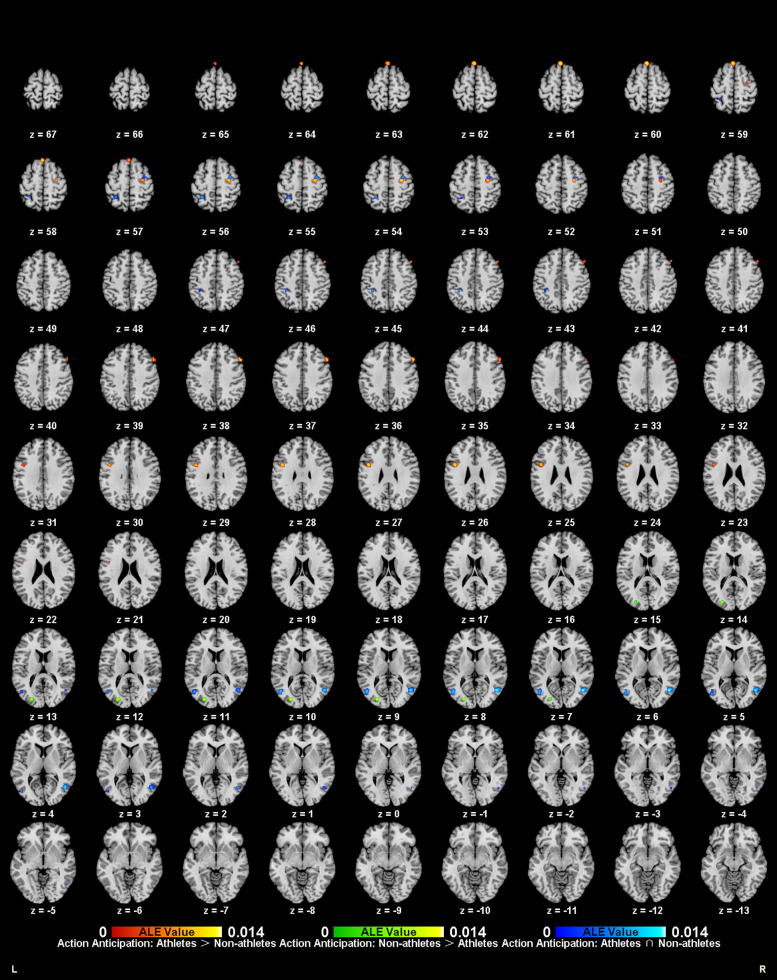
Common and specific brain activation in athletes and non-athletes during action anticipation. Red-yellow marks indicated clusters where athletes exhibited greater activation than non-athletes. Green-yellow marks indicated clusters where non-athletes exhibited greater activation than athletes. Blue-cyan marks indicated clusters where athletes and non-athletes had overlapped activation.

Meta-analytic calculations of 89 reported brain region coordinates with significantly stronger activation in athlete groups across six studies identified four significant activation clusters, concentrated in the left superior frontal gyrus (SFG BA6), bilateral precentral gyrus (PreCG BA6), and right middle frontal gyrus (MFG BA8) (Table 5, Figure 5).

**Table 5:** Conjunction and contrast analyses between athletes and non-athletes during action anticipation.

Cluster	Volume	Brain regions	Hemisphere	Brodmann area	MNI coordinates			ALE (×10^−2^)
					X Y Z	Y	Z	
Action anticipation: athletes > non-athletes								
1	488	Superior Frontal Gyrus	L	6	−2	32	60	1.43
2	400	Precentral Gyrus	R	6	28	−10	54	1.24
3	384	Precentral Gyrus	L	6	−44	4	26	1.27
4	336	Middle Frontal Gyrus	R	8	54	22	38	1.20
Action anticipation: non-athletes > athletes								
1	400	Middle Occipital Gyrus	L	17	−24	−84	10	1.17
Action anticipation: athletes ∩ non-athletes								
1	752	Middle Temporal Gyrus	R	37	46	−64	6	1.45
2	496	Middle Temporal Gyrus	L	37	−46	−66	8	1.13

Meta-analytic calculations of 148 reported brain region coordinates with significantly stronger activation in control groups across seven studies identified one activation cluster, concentrated in the left middle occipital gyrus (MOG BA17) (Table 5, Figure 5).

To determine common activation regions between athletes and non-athletes during action anticipation tasks, a conjunction analysis was performed on the individual ALE results of the two groups. Findings showed common activation of the bilateral middle temporal gyrus (MTG BA37) in both groups (Table 5, Figure 5).

### 3.4 Conjunction and contrast analyses between motor imagery and action anticipation

Pairwise conjunction and contrast analyses of brain activity were performed between motor imagery and action anticipation in athletes and non-athletes.

#### 3.4.1 Comparison between motor imagery and action anticipation in athletes

**Figure 6:**
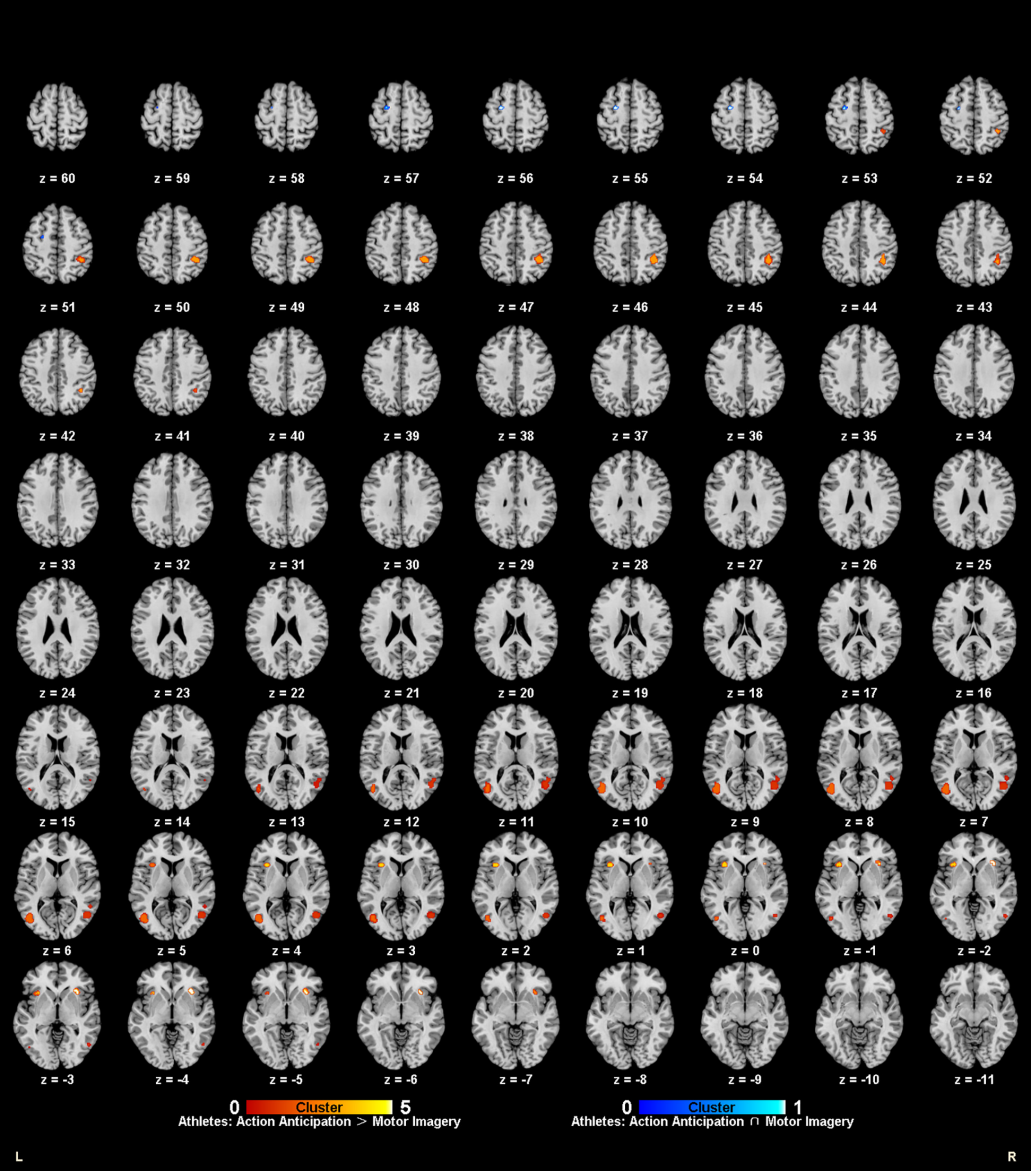
Common and specific brain activation in action anticipation and motor imagery in athletes. Red-yellow marks indicated clusters where athletes showed greater activation in action anticipation than in motor imagery tasks. Blue-cyan marks indicated clusters where athletes showed common activation in both action anticipation and motor imagery tasks.

To explore the differences and commonalities in brain regions activated by different task categories, this study performed contrast and conjunction analyses on the ALE maps of athletes under the two tasks (Table 6, Figure 6). Athletes showed common activation of the left middle frontal gyrus (MFG BA6) in both motor imagery and action anticipation tasks. Additionally, athletes exhibited significantly more activation in the right middle temporal gyrus (MTG BA39), left inferior temporal gyrus (ITG BA37), right inferior parietal lobule (IPL BA40), left insula (BA13) and right claustrum during action anticipation tasks compared to motor imagery tasks. No brain regions showed greater activation in motor imagery tasks than in action anticipation tasks among athletes.

**Table 6:** Conjunction and contrast analyses between motor imagery and action anticipation in athletes.

Cluster	Volume	Brain regions	Hemisphere	Brodmann area	MNI coordinates			*p* (×10^−2^)
					X	Y	Z	
Athletes: action anticipation > motor imagery								
1	1,568	Middle Temporal Gyrus	R	39	49.4	−61.2	9.5	0.3
		Superior Temporal Gyrus	R	39	54	−54	8	0.5
		Middle Occipital Gyrus	R	37	51	−68	6	0.7
		Middle Temporal Gyrus	R	37	47.3	−61.3	3.3	1
2	1,504	Inferior Temporal Gyrus	L	37	−46.3	−68	3	0.2
		Middle Temporal Gyrus	L	37	−45	−69.5	9.1	0.6
3	984	Inferior Parietal Lobule	R	40	40	−36	44	1.7
		Inferior Parietal Lobule	R	40	38.4	−43.7	47.1	2.1
		Inferior Parietal Lobule	R	40	38	−50.7	44	3.9
4	432	Insula	L	13	−34	16	4	3.5
		Insula	L	13	−35	20	0	3.6
5	296	Claustrum	R	/	34	19	−1	1.2
		Claustrum	R	/	30.7	21.7	−3.5	2.3
Athletes: motor imagery > action anticipation								
-	-	-	-	-	-	-	-	-
Athletes: action anticipation ∩ motor imagery								
1	160	Middle Frontal Gyrus	L	6	−24	−6	54	/

#### 3.4.2 Comparison between motor imagery and action anticipation in non-athletes

**Figure 7:**
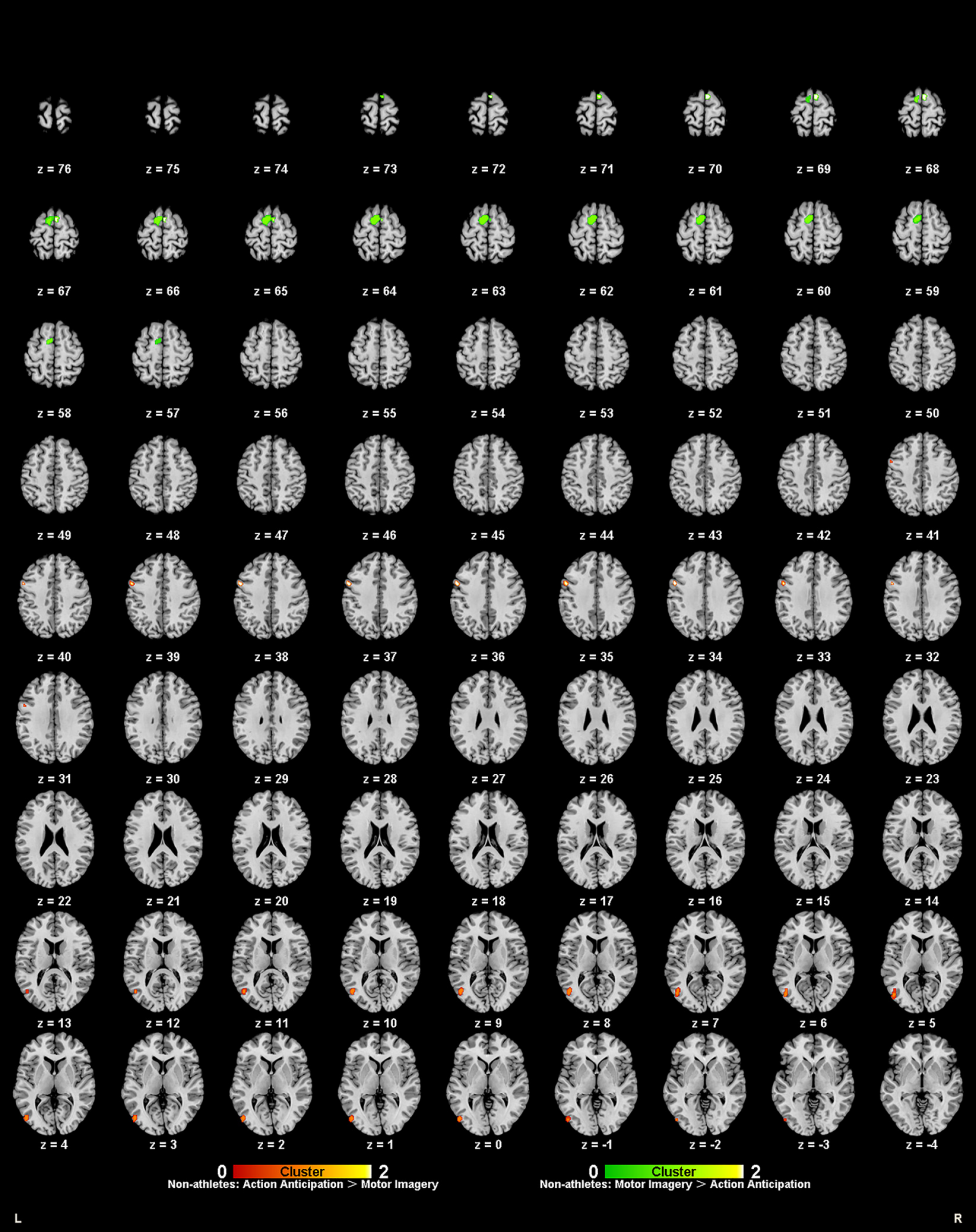
Common and specific brain activation in action anticipation and motor imagery in non-athletes. Red-yellow marks indicated clusters where non-athletes showed greater activation in action anticipation than in motor imagery tasks. Green-yellow marks indicated clusters where non-athletes showed greater activation in motor imagery than in action anticipation tasks.

To explore the differences and commonalities in brain regions activated by different task categories, this study performed contrast and conjunction analyses on the ALE maps of non-athletes under the two tasks (Table 7, Figure 7). Non-athletes showed no common brain activation between motor imagery and action anticipation tasks. During action anticipation tasks, non-athletes exhibited significantly more activation in the left inferior temporal gyrus (ITG BA19) and left precentral gyrus (PreCG BA6) compared to motor imagery tasks. Conversely, during motor imagery tasks, non-athletes showed greater activation in the left medial frontal gyrus (MedFG BA6) and left superior frontal gyrus (SFG BA6) than in action anticipation tasks.

**Table 7:** Conjunction and contrast analyses between motor imagery and action anticipation in non-athletes.

Cluster	Volume	Brain regions	Hemisphere	Brodmann area	MNI coordinates			*p* (×10^−2^)
					X	Y	Z	
Non-athletes: action anticipation > motor imagery								
1	840	Inferior Temporal Gyrus	L	19	−48.2	−73.8	3.8	2.2
		Middle Temporal Gyrus	L	37	−46.8	−67.6	12	4.1
2	288	Precentral Gyrus	L	6	−54	2	38	1.1
		Precentral Gyrus	L	6	−52	4	34	1.6
Non-athletes: motor imagery > action anticipation								
1	1,584	Medial Frontal Gyrus	L	6	−2.7	6.4	64.2	0.7
		Medial Frontal Gyrus	L	6	−8	8	63	0.8
		Superior Frontal Gyrus	L	6	−6	3	64	1.4
		Medial Frontal Gyrus	L	6	−5	3	58	2.1
		Medial Frontal Gyrus	L	6	−9.6	−0.9	63.2	1.4
		Superior Frontal Gyrus	L	6	−8	−8	66	2.5
2	352	Superior Frontal Gyrus	R	6	2.6	4.3	70.3	0.9
		Medial Frontal Gyrus	R	6	2	0	67	2
Non-athletes: action anticipation ∩ motor imagery								
—	—	—	—	—	—	—	—	/

### 3.5 MACM analysis between groups

We performed MACM analysis on the ROIs extracted from the ALE analysis between athletes and non-athletes. In the motor imagery task, two ROIs were selected from the comparison where athletes showed greater activation than non-athletes. As no coordinate points exhibited greater activation in non-athletes than in athletes, and there were no commonly activated brain regions between the two groups, MACM analysis was not conducted. In the action anticipation task, four ROIs were selected from the comparison where athletes showed greater activation than non-athletes, and two ROIs were selected for the conjunction analysis of activation between the two groups. Since no coordinate points showed greater activation in non-athletes than in athletes, MACM analysis was also not performed.

#### 3.5.1 Motor imagery: athletes > non-athletes

**Figure 8:**
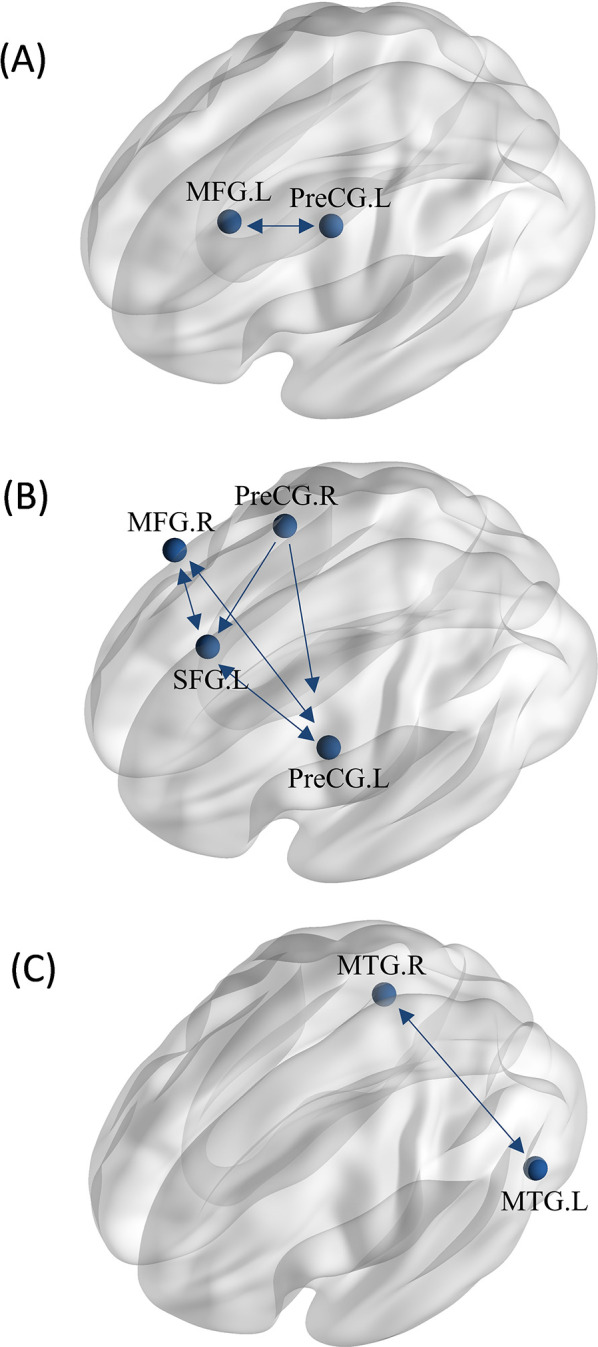
MACM analysis between groups. **(A)** The MACM map of athletes exhibiting greater activation than non-athletes during motor imagery. **(B)** The MACM map of athletes exhibiting greater activation than non-athletes during action anticipation. **(C)** The MACM map of common activation in athletes and non-athletes during action anticipation. (MFG.L, left middle frontal gyrus; PreCG.L, left precentral gyrus; MFG.R, right middle frontal gyrus; PreCG.R, right precentral gyrus; SFG.L, left superior frontal gyrus; MTG.L, left middle temporal gyrus; MTG.R, right middle temporal gyrus. Bidirectional arrow represent bidirectionality, indicating that the variance in two nodes is predictive of each other. Unidirectional arrow represent unidirectionality, indicating that variance in one node is predictive of variance in another, but not vice versa.).

For greater activation in athletes during motor imagery tasks, two ROIs were extracted from the ALE analysis for coactivation mapping analysis. We found significant bidirectional functional connectivity between the left middle frontal gyrus (MFG) and left precentral gyrus (PreCG) (Figure 8A).

#### 3.5.2 Action anticipation: athletes > non-athletes

For greater activation in athletes during action anticipation tasks, four regions of interest (ROIs) were extracted from the ALE analysis for coactivation mapping analysis. We found significant bidirectional functional connectivity between the left precentral gyrus (PreCG), left superior frontal gyrus (SFG), and right middle frontal gyrus (MFG). Additionally, significant unidirectional functional connectivity was observed between the right precentral gyrus (PreCG)and left precentral gyrus (PreCG), as well as between the right precentral gyrus and left superior frontal gyrus (Figure 8B).

#### 3.5.3 Action anticipation: athletes ∩ non-athletes

For common activation in both groups, two ROIs were extracted from the ALE analysis for coactivation mapping analysis. We found significant bidirectional functional connectivity between the right middle temporal gyrus (MTG) and left middle temporal gyrus (MTG) (Figure 8C).

### 3.6 MACM analysis between tasks

MACM analysis was performed on the ROIs extracted from the conjunction and contrast analyses between motor imagery and action anticipation. In the athlete group, five ROIs were selected from the comparison where brain activation during action anticipation was greater than that during motor imagery. As no brain regions showed greater activation during motor imagery than during action anticipation, and only one brain region was commonly activated in the two tasks, MACM analysis was not performed. In the non-athlete group, two ROIs were selected from the comparison where brain activation during action anticipation was greater than that during motor imagery, and two ROIs were selected from the analysis of brain regions with greater activation during motor imagery than during action anticipation. Since no commonly activated brain regions were found between the two tasks, MACM analysis was not performed.

#### 3.6.1 Athletes: action anticipation > motor imagery

**Figure 9:**
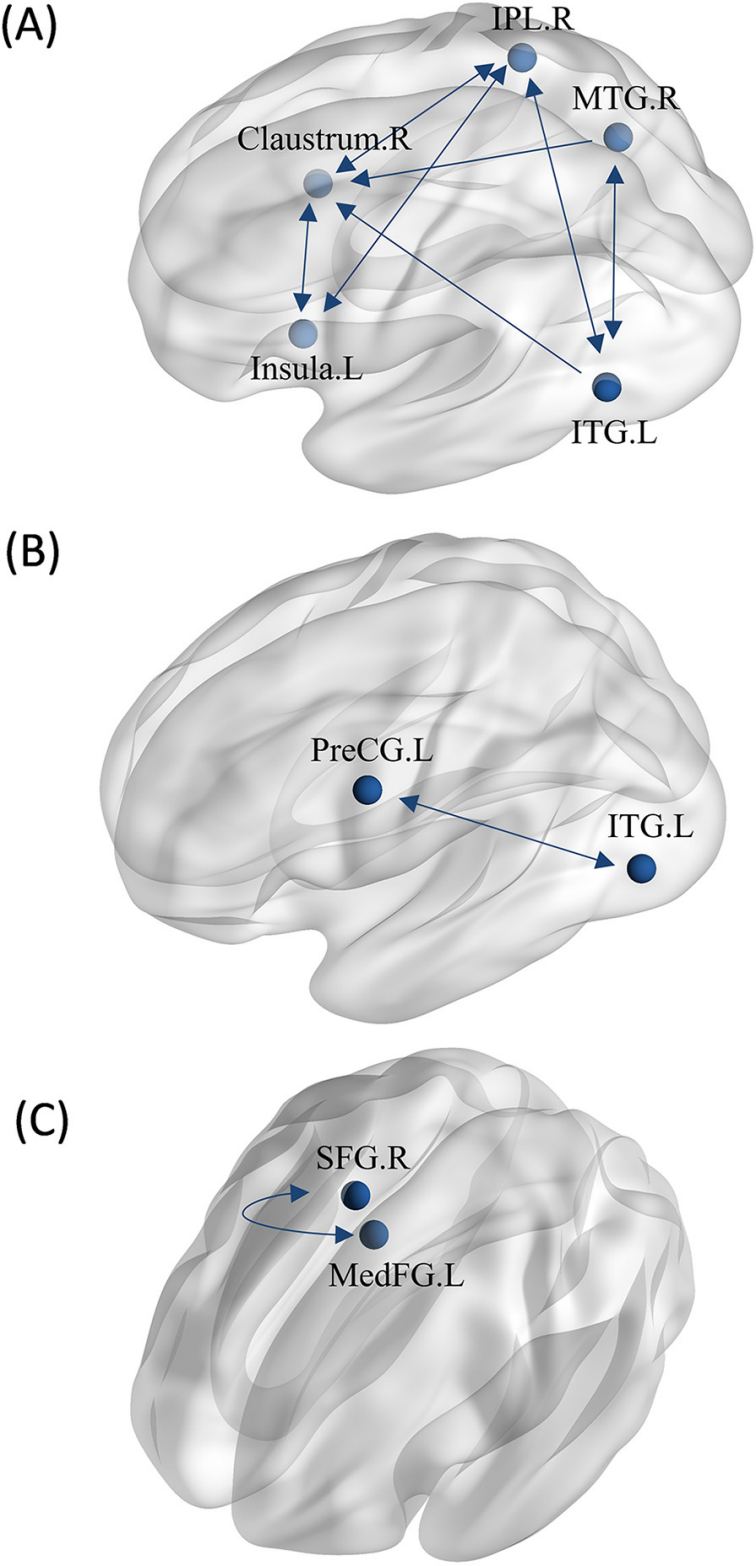
MACM analysis between tasks. **(A)** The MACM map of athletes revealed greater activation during action anticipation compared to motor imagery. **(B)** The MACM map of non-athletes showed greater activation during action anticipation than during motor imagery. **(C)** The MACM map of non-athletes showed greater activation during motor imagery than during action anticipation. (IPL.R, right inferior parietal lobule; ITG.L, left inferior temporal gyrus; MTG.R, right middle temporal gyrus; PreCG.L, left precentral gyrus; SFG.R, right superior frontal gyrus; MedFG.L, left medial frontal gyrus; Bidirectional arrow represent bidirectionality, indicating that the variance in two nodes is predictive of each other. Unidirectional arrow represent unidirectionality, indicating that variance in one node is predictive of variance in another, but not vice versa.).

Regions with greater activation in action anticipation tasks compared to motor imagery tasks were identified as ROIs for MACM analysis, resulting in a total of five ROIs. We found significant bidirectional functional connectivity between the right middle temporal gyrus (MTG) and left inferior temporal gyrus (ITG), between the right inferior parietal lobule (IPL) and left inferior temporal gyrus (ITG), There are bidirectional functional connections between each pair of the left insula, right claustrum, and right inferior parietal lobule (IPL). Additionally, significant unidirectional functional connectivity was observed between the right claustrum and right middle temporal gyrus (MTG), as well as between the right claustrum and left inferior temporal gyrus (ITG) (Figure 9A).

#### 3.6.2 Non-athletes: action anticipation > motor imagery

Regions with greater activation in action anticipation tasks compared to motor imagery tasks were identified as ROIs for MACM analysis, resulting in a total of two ROIs. We found bidirectional functional connectivity between left inferior temporal gyrus (ITG) and left precentral gyrus (PreCG) (Figure 9B).

#### 3.6.3 Non-athletes: motor imagery > action anticipation

Regions with greater activation in motor imagery tasks compared to action anticipation tasks were identified as ROIs for MACM analysis, resulting in a total of two ROIs. We found bidirectional functional connectivity between the right superior frontal gyrus (SFG.R) and left medial frontal gyrus (MedFG.L) (Figure 9C).

## 4 Discussion

We conducted meta-analyses to determine the neural mechanisms underlying superior athletic performance by comparing the blood oxygen level-dependent (BOLD) signals of athletes and non-athletes in specific regions during motor imagery and action anticipation. Moreover, we used connectivity models to demonstrate how athletes develop unique spatial-topographically nested regions between motor imagery and action anticipation. Our main findings are: (i) during motor imagery tasks, athletes exhibited greater activation in the left middle frontal gyrus and left precentral gyrus compared to non-athletes; (ii) during action anticipation tasks, athletes showed greater activation in the left superior frontal gyrus, bilateral precentral gyrus, and right middle frontal gyrus compared to non-athletes and non-athletes showed greater activation in the left middle occipital gyrus compared to athletes; (iii) overlap in left middle frontal gyrus, athletes activated extra brain regions during action anticipation compared to motor imagery tasks while non-athletes have distinct activation patterns in two tasks; (iv) beyond motor imagery, athletes developed functional connectivity in right middle temporal gyrus, left inferior temporal gyrus, right inferior parietal lobule, right insula and right claustrum during action anticipation, which was not found in non-athletes.

### 4.1 Athletes’ neural advantage in motor imagery

The results of the ALE meta-analysis showed that athletes exhibited greater activation in the left MFG (BA8) and left PreCG (BA6) during motor imagery tasks compared to non-athletes. Aligning with the previous studies (23, 26), motor experts showed stronger activation in the PreCG. Housing the primary motor cortex, PreCG is consistently activated during both actual movement and motor imagery (54). The stronger activation in athletes may reflect enhanced mental rehearsals of the observed domain-specific stimuli. The MFG is more involved in the higher-order processing aspects of motor imagery (54). The stronger activation found in athletes may represent the early readiness in perceptual processing for action execution. In terms of hemispheric dominance, athletes' higher involvement of AON only appeared in the left hemisphere, which is consistent with the finding that the left hemisphere is dominant for complex motor sequences (55).

In contrast, non-athletes exhibited no significant activation compared to athletes, suggesting that imagery performance in novices may rely on less efficient or more variable neural strategies. The conjunction analysis further identified common activation in the left MedFG (BA6), highlighting a shared reliance on medial premotor areas during imagery across groups. These findings align with prior observations in motor expertise literature (56), which suggest that training sharpens the efficiency of AON circuits during motor imagery.

The functional connectivity patterns revealed in the MACM analysis—bidirectional coupling between MFG and PreCG—further emphasize a streamlined simulation network in athletes, consistent with the neural efficiency hypothesis (19). Compared to the broader, more distributed activation patterns often seen in novices. Both the PreCG and MFG are key components of the AON, with the PreCG involved in motor aspects (57, 58) and the MFG contributing to higher-level cognitive processing during action observation (59, 60). The left-hemisphere-dominant MFG—PreCG network suggests that expertise fosters more specialized and automatic engagement of AON—related regions during imagery.

### 4.2 Athletes’ neural advantage in action anticipation

The results of the ALE meta-analysis showed that athletes exhibited greater activation in the left SFG (BA6), bilateral PreCG (BA6), and right MFG (BA8) during action anticipation tasks compared to non-athletes. These regions collectively form a core part of the AON (61), supporting more goal-oriented actions driven by mirror neurons (62). The robust activation of bilateral PreCG (BA4/6) and frontal regions in athletes likely reflects superior predictive modeling abilities honed through repeated sport-specific anticipation training (63). In contrast, novices showed greater activation in the left MOG (BA17), indicating a more substantial reliance on visual processing pathways rather than motor simulation, consistent with findings from a prior study (24). The conjunction analysis further revealed bilateral MTG (BA37) activation in both groups, highlighting its significant role as a multimodal hub integrating visual and motor information (64).

The MACM results in athletes demonstrated a tightly interconnected network between left PreCG, left SFG, and right MFG, suggesting that expertise fosters efficient fronto-motor loops for rapid action prediction—a hallmark of optimized AON engagement. These patterns parallel earlier findings in motor imagery tasks, where athletes displayed more selective and efficient neural recruitment, while novices engaged broader, less specialized cortical resources (19).

In summary, left-dominant functional interactions in AON are enhanced during motor imagery tasks after extensive motor experience. Meanwhile, the AON network is refined during action anticipation, supporting more effective anticipation of complex action sequences. Together, these results underscore that athletes developed more focused brain networks for complex motor tasks.

### 4.3 Athletic experience-driven nested simulation network

The meta-analysis of motor imagery and action anticipation tasks revealed distinct yet partially overlapping neural activation patterns in athletes and non-athletes. During motor imagery, athletes exhibited stronger activation in regions such as the MFG (BA6), SFG (BA6), bilateral PreCG (BA6/4), IFG (BA44), left ITG (BA37) and right STG (BA42), while action anticipation elicited enhanced broader activation in the MOG (BA37), MTG (BA37), STG (BA39), IPL (BA40), bilateral PreCG (BA6), claustrum, and precuneus (BA7). In contrast, non-athletes relied more heavily on posterior sensory areas (MOG BA37, ITG BA37) and the IPL (BA40) during action anticipation, while their motor imagery predominantly engaged medial and superior frontal areas (MFG BA6, SFG BA6) and subcortical structures (lentiform nucleus). Together, athletes depend highly on motor imagery to perform superior action anticipation, demonstrating an integrated sensorimotor simulation network during both anticipation and imagery tasks, whereas non-athletes engage distinct, task-dependent networks with greater reliance on visual and higher-order cognitive regions.

Interestingly, MACM analyses further underscored these differences in network organization. In athletes, action anticipation relative to motor imagery involves enhanced bidirectional connectivity between the left insula, right IPL, and right claustrum. The insula and claustrum frequently co-activate with the IPL in networks underlying attention and interoception (awareness of internal bodily states) (65). Athletic action anticipation demonstrates a remarkable ability to predict their opponent's movements, enabling them to react swiftly with their own physical responses. During the process, intensive attention is required to external and internal information. The result suggests long term athletic training improve brain connectivity supporting the ability to extract cognitive and bodily information, which may act as key components for superior action anticipation beyond motor imagery. In addition, athletes formed stronger bidirectional connectivity between left ITG and right IPL, left ITG and right MTG when performing the action anticipation task compared to the motor imagery task. In line with previous studies, the right IPL and right MTG are implicated in motor control and attentional efficiency, both crucial for athletic performance (66, 67). The current study confirmed the interaction between left ITG and the above two areas, suggesting visual processing is essential for athletic action anticipation. Conversely, non-athletes exhibited a more visually anchored network, with bidirectional coupling in temporal lope (ITG—PreCG) during action anticipation, and a prefrontal loop (SFG—MFG) during motor imagery. These findings suggest that athletic expertise fosters the development of an integrated MNS-AON network, not only interact visual information with higher order cognitive process, but also effectively extracting internal and external information.

The current study has several limitations. First, all included studies employed cross-sectional designs, which restrict the ability to draw causal inferences about the effects of long-term training on brain activation. Without longitudinal data, it remains unclear whether the observed neural differences reflect training-induced plasticity or pre-existing traits. Second, although the number of included studies meets the minimum requirement for ALE stability, the relatively small sample size for specific task categories (e.g., action anticipation, motor imagery) may limit statistical power and generalizability. Additionally, the current study used a more exploratory analytic ALE method and may increase the risk of false positives.

Together, our findings show that in athletes, the neural circuits supporting action anticipation are spatial-topographically nested within and dynamically coupled with those engaged during motor imagery. Specifically, bilateral PreCG, IPL, and temporal regions (MTG/ITG) form a core simulation network that flexibly supports both internal rehearsal and predictive processing of actions. This nested organization likely reflects an experience-driven optimization of AON/MNS-related circuits (14, 68), enabling athletes to efficiently transition between motor imagery and anticipatory states. In contrast, non-athletes demonstrate more segregated, task-specific activation patterns, suggesting less integrated sensorimotor representations and reduced efficiency in cross-contextual action simulation. Future research may explore how this nested simulation network evolves across different stages of skill acquisition and whether targeted interventions can enhance its efficiency in novice populations.
