# Supplementary figures and images for: Brain network of athletes in motor imagery and action anticipation: an ALE meta-analysis and MACM analysis
Source: Front Sports Act Living. 2025 Aug 25;7:1652165. doi: 10.3389/fspor.2025.1652165 (PMC12415032; doi:10.3389/fspor.2025.1652165)

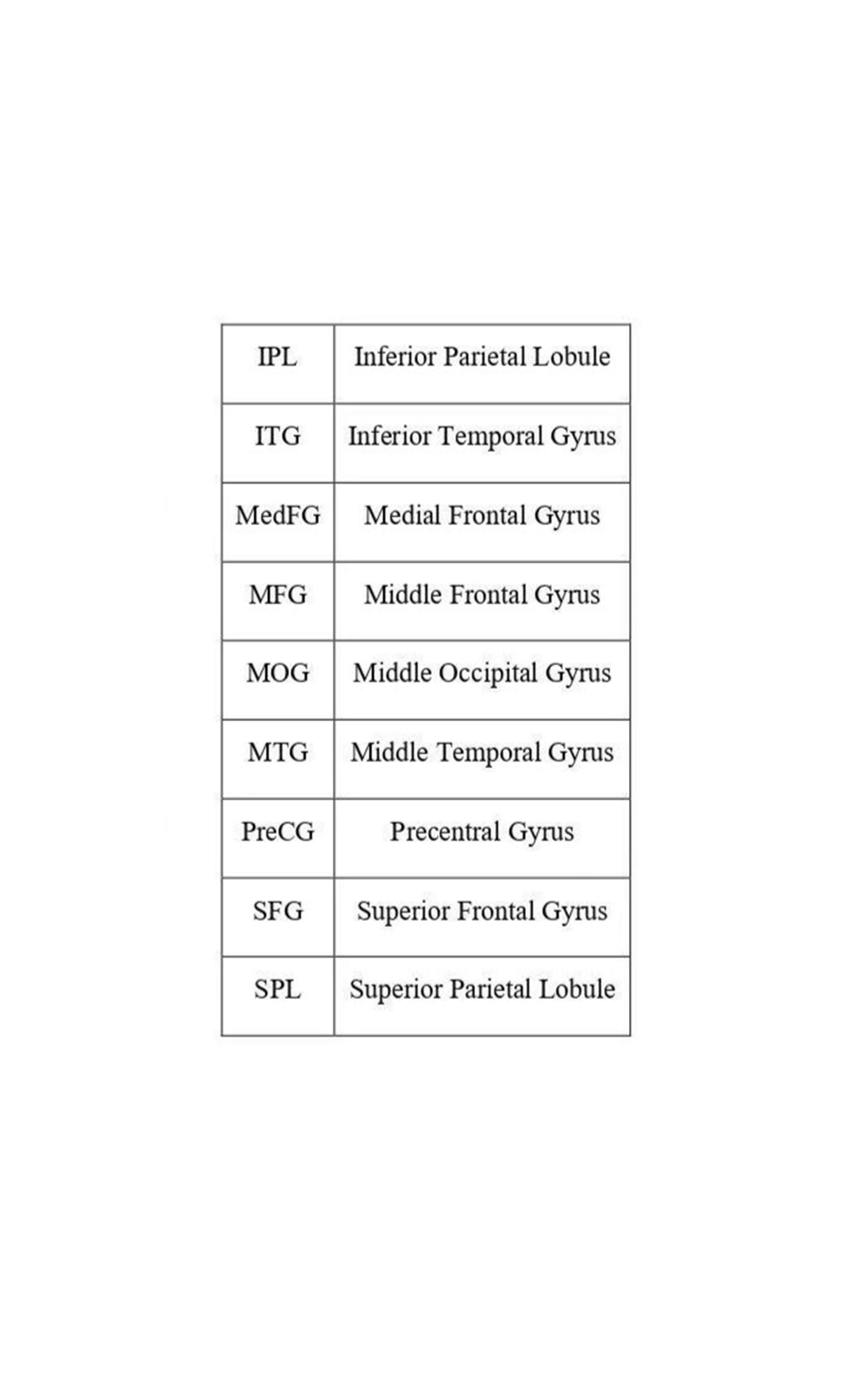

Supplement: Supplementary file 1 [file Image1.png]

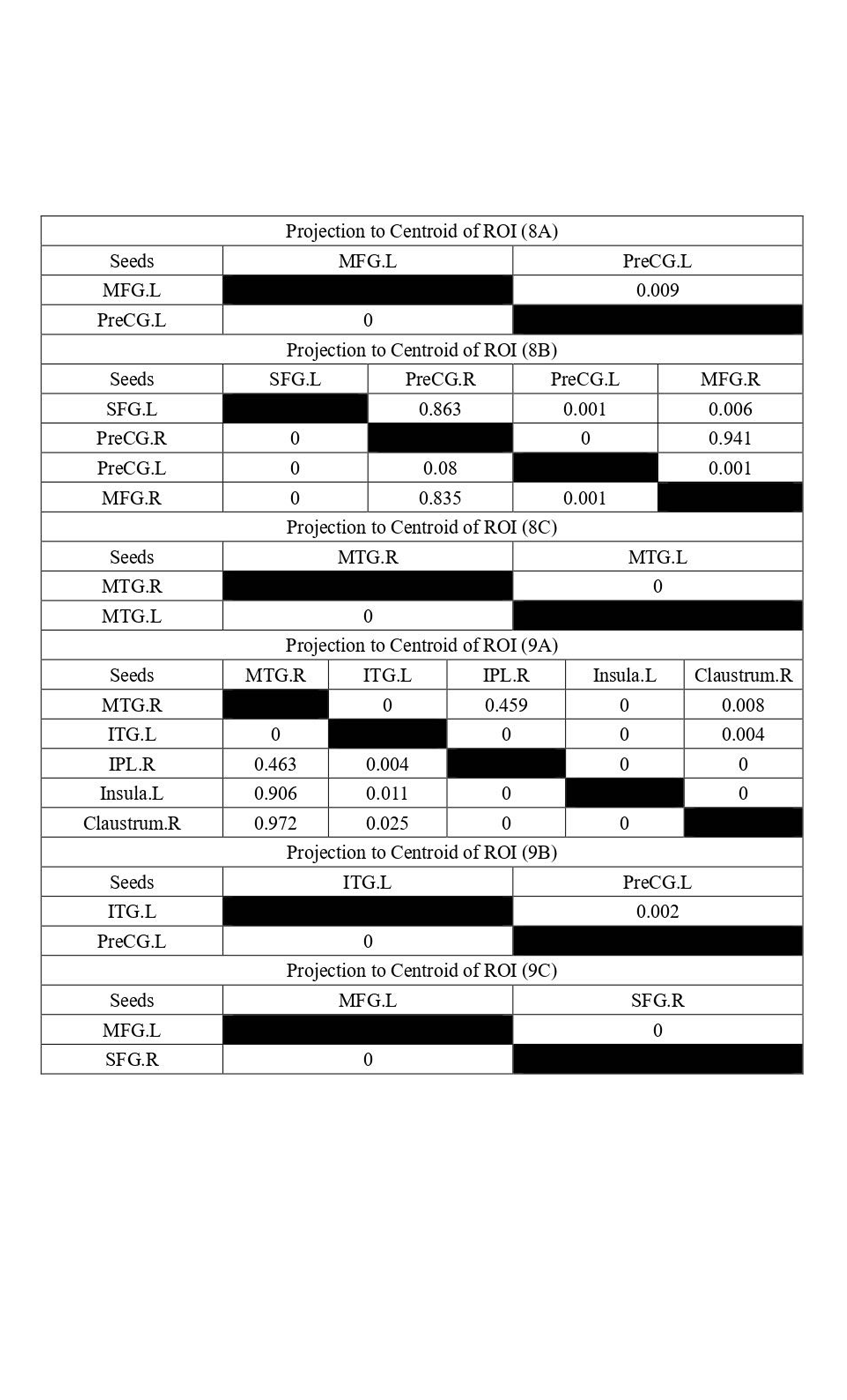

Supplement: Supplementary file 2 [file Image2.png]
